# Temephos susceptibility status in *Aedes aegypti* populations from western, central, and eastern regions of Cuba

**DOI:** 10.1186/s13071-025-06955-0

**Published:** 2026-02-14

**Authors:** Luis Augusto Piedra, Dayana Rodriguez, Waldemar Baldoquin, Pablo Cardenas, Eric Camacho, Israel Garcia, Ilien Mitjans, Nell Cox, Veerle Vanlerberghe, Maria del Carmen Marquetti

**Affiliations:** 1 Institute of Tropical Medicine “Pedro Kouri”, Havana, Cuba; 2 Institute of Tropical Medicine Antwerp, Antwerp, Belgium

**Keywords:** Insecticide, Resistance, *Aedes aegypti*, Temephos

## Abstract

**Background:**

*Aedes aegypti* chemical control remains the major strategy to prevent dengue, Zika, and Chikungunya outbreaks. The Cuba archipelago organizes a constant surveillance in its entire territory to follow up the distribution and infestation levels, but also insecticide resistance of *Ae. aegypti*, to estimate arbovirus transmission risk for its inhabitants. The objective of this study was to determine temephos susceptibility status in the *Ae. aegypti* population from western, central, and eastern regions of Cuba.

**Methods:**

*Aedes* larvae bioassays were performed following World Healthy Organization (WHO) methodology. Entomological samples were collected in the western (Pinar del Rio and Matanzas), central (Santa Clara [Villa Clara], Cienfuegos, Sancti Spíritus, and Camagüey), and eastern (Las Tunas, Holguín, Santiago de Cuba, and Guantánamo) regions of Cuba from February 2022 to June 2023.

**Results:**

This study showed susceptibility in Las Tunas population (RR_50_ = 0.94). Santa Clara [Villa Clara] (RR_50_ = 8.94), Cienfuegos (RR_50_ = 5.88), and Sancti Spíritus (RR_50_ = 6.7) populations showed moderate resistance and the rest showed high resistance (RR_50_ > 10) to temephos.

**Conclusions:**

Most *Ae. aegypti* populations tested from western, central and eastern regions of Cuba showed spatial homogeneity of temephos resistance owing to the intensive use of this larvicide since 1981. Insecticide resistance management by the National Vector Control Program is required to reverse temephos resistance development.

**Graphical abstract:**

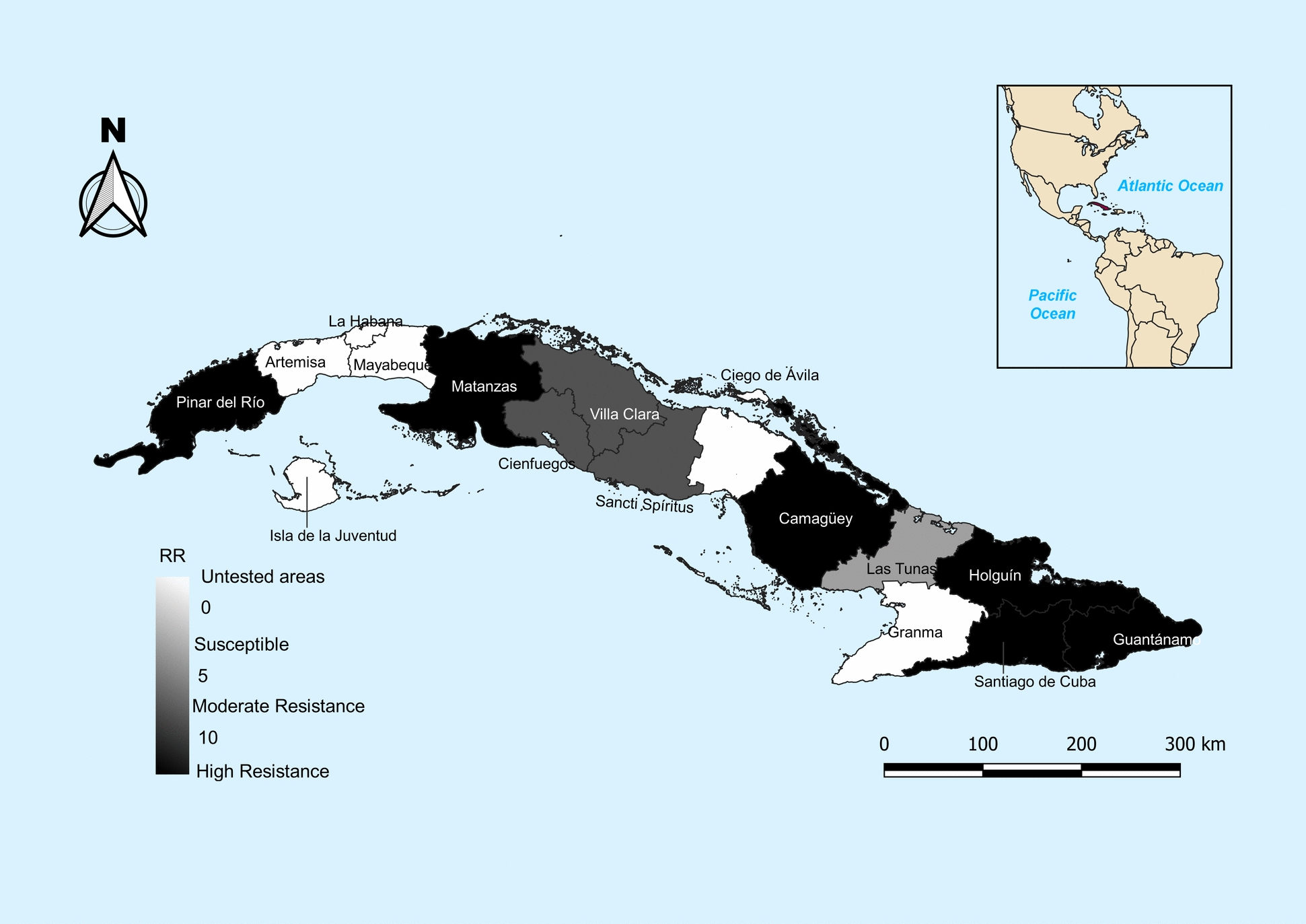

## Background

The world has experienced an increase in arbovirus transmission, mainly dengue, but also for Zika, Chikungunya and yellow fever since 2000. Recent investigations estimate that approximately 2.5 billion people live in countries where dengue fever is endemic, with an estimate by the World Health Organization (WHO) of 100–400 million infections annually [1]. These diseases are mainly transmitted by the widespread mosquito *Aedes (Stegomyia) aegypti* (Linnaeus, 1762), with the second most important vector, *Aedes albopictus*.

In view of the absence of available vaccines for Chikungunya and Zika, and the limited availability of dengue vaccines, mosquito control continues to be the main alternative to prevent and control arbovirus transmission in human populations, with chemical insecticide application recognized globally as the most used tool to combat outbreaks and epidemics [2]. Among the diseases considered a public health problem worldwide, with the highest use of insecticides in spraying operations (i.e., residual spraying, space spraying, larvicides), dengue uses 22.9% with respect to malaria (60.8%), leishmaniasis (9.7%), and Chagas (4.8%) [3]. Larvicides account for 33.2% and 34.3% of insecticides used in spray operations of dengue vector control programs in the Asia-Pacific and Latin America, and Caribbean regions, respectively [3]. Temephos, an organophosphate insecticide, is the most widely used larvicide to control *Ae. aegypti* [4–6]. Its intensive use has generated insecticide resistance in different *Ae. aegypti* populations associated with the metabolic action of enzymes, esterases, and glutathione S-transferases in Latin America [7–10] and Asia [11]. The development of temephos resistance in *Ae. aegypti* populations has affected the effectiveness of dengue control programs in some countries such as Mexico [12], Brazil [13] and Cambodia [14] where this insecticide has been the most widely used for mosquito larvae control.

In Cuba, temephos has been used for larviciding of mosquitoes by the National Vector Control Program of *Ae. aegypti* since 1981. Nowadays, it is still intensively used in Cuba, depending on the epidemiological patterns, hence the need to have precise estimates of its resistance distribution. The first report of temephos resistance in *Ae. aegypti* populations came from the eastern part of Cuba in 1997 [15], with punctual reports of Santiago de Cuba in 1997–2007 [9, 16–18], Pinar del Rio in 2014–2015 [19], and Havana in 2001–2018 [9, 20–25]. However, there is no detailed description of the temephos resistance status in Cuban *Ae. aegypti* populations at a national scale, which is needed to guide insecticide management and implement more efficient vector control strategies. The objective of this study was to monitor temephos susceptibility and its space–time distribution in mosquito populations from different regions of Cuba.

## Methods

### Settings

Cuba, one of the 13 islands of the Caribbean Sea, is organized into 15 provinces and a special municipality (Isla de La Juventud), with Havana as its capital and most populated city. It is the third most populated country in the Antilles, with an area of 109,884 km^2^ and a population of 11,089,511 in 2022 according to the National Office of Statistics and Information from Cuba.

### *Aedes aegypti* colonies

*New Orleans strain* *Ae. aegypti*, the susceptible strain used, was supplied by the Pasteur Institute of Guadalupe, France.

### Field population

Mosquito collection was carried out in several provincial capitals from the western (Pinar del Rio and Matanzas), central (Santa Clara, Cienfuegos, Sancti Spíritus, and Camagüey), and eastern (Las Tunas, Holguín, Santiago de Cuba, and Guantánamo) regions of Cuba (Table 1). These locations were selected taking into account their high population density and dengue transmission patterns.

**Table 1:** Geographical data of sites where *Aedes aegypti* populations were collected to evaluate their temephos resistance status

Cuban regions	Provinces	Municipality	Coordinates	Area (km^2^)
Western	Pinar del Rio	Pinar del Rio	22° 24′ 44″ N 83° 40′ 19″ W	691.12
	Matanzas	Matanzas	23° 02′ 58″ N 81° 34′ 25″ W	317
Central	Villa Clara	Santa Clara	22° 24′ 24″ N 79° 57′ 11″ W	61.82
	Cienfuegos	Cienfuegos	22° 08′ 44″ N 80° 26′ 11″ W	4180.02
	Sancti Spíritus	Sancti Spíritus	21° 56′ 02″ N 79° 26′ 38″ W	1147
	Camagüey	Camagüey	21° 23′ 2″ N 77° 54′ 27″ W	1106
Eastern	Las Tunas	Las Tunas	20° 57′ 25″ N 76° 57′ 3″ W	895.34
	Holguín	Holguín	20° 53′ 14″ N 76° 15′ 47″ W	655.9
	Santiago de Cuba	Santiago de Cuba	20° 01′ 18″ N 75° 49′ 46″ W	1023.8
	Guantánamo	Guantánamo	22° 24′ 44″ N 83° 40′ 19″ W	741

### Mosquito samples collection

Mosquito eggs were collected from ovitraps, which were placed every 100 m in the center of the provincial capitals, from February to August 2022 and from January to June 2023. The paper strips with mosquito eggs were transported to the Vector Control Department in the Institute of Tropical Medicine Pedro Kouri, where the *Aedes* eggs were reared into adults for morphological identification [26]. Adult mosquitoes were transferred to cages (30 × 30 × 30 cm) and maintained on a 10% sucrose solution in the following environmental conditions: 12/12 h light/dark cycle, 27 ± 1 °C, and 70–80% relative humidity. Wild *Ae. aegypti* females were allowed to feed with blood for 1 h through membrane feeding (Hemotek). In each cage, we placed a beaker with water and a paper strip inside the edge to ensure the collection of eggs laid by females. Egg strips from the F1 generation of each field mosquito colony were placed on a wet surface in trays for 24 h to allow proper embryogenesis. Once removed from trays, paper strips were left to dry. Subsequently, F1 egg strips were hatched in trays with 2.5 L of warm water. Mosquito larvae were fed fishmeal and maintained under the conditions described above. Pupae were transferred to the cages and grown into adults to begin the vector’s life cycle again [27].

### Larval bioassays

Insecticide resistance status of temephos (92% of purity; supplied by CPHR, Cuba) was evaluated in larvae bioassays according to WHO methodology [28]. A range of concentrations (0.1, 0.5, 1.0, 1.5, and 2.0 ppm) were tested with four replicates each and one control per concentration. A total of 25 late third instar and/or early fourth instar larvae from F1 generation, of uniform size, were placed in each plastic cup. Cups of all replicates contained 99 ml of tap water and 1 ml of prepared insecticide solution. Controls cups were treated using 1 ml of the solvent (acetone) used to prepare temephos concentrations. Each of the insecticide concentrations tested, prepared in standard (weight/volume) acetone were replicated five times, then 100 larvae were evaluated by each concentration and, hence, 500 larvae were assayed for each bioassay. All *Ae. aegypti* larvae were reared and maintained at 12/12 h light/dark cycle, 27 ± 1 °C, and 70–80% relative humidity. Mortality was determined 24 h after application of the insecticide.

### Data analysis

The results of larvae bioassays were analyzed using the probit test implemented by SPSS Statistics Program version 21. The Resistance ratio (RR_50_/RR_90_) was calculated by comparing the value of lethal concentration (LC_50_/LC_90_) of the field colonies with New Orleans strain. Mosquito populations were classified as resistant or susceptible using the criteria (RR ≤ 5: susceptible, 5 < RR ≤ 10: moderate resistance, and RR > 10: high resistance) [29]. Mapping of spatial distribution to temephos susceptibility status in Cuban *Ae. aegypti* populations was done using QGIS Geographic Information System version 3.34.12.

## Results

The resistance ratios (RR_50_/RR_90_) showed temephos resistance in most Cuban *Ae. aegypti* populations tested (Table 2).

**Table 2:** Lethal Concentrations (LC_50_/LC_90_) of temephos resistance in *Ae. aegypti* populations of Cuba

Cuban regions	*Ae. aegypti* colonies	^1^*N*	^2^LC_50_ (ppm)	^3^RR_50_	^2^LC_90_ (ppm)	^3^RR_90_	^4^b (± SD)	^5^ χ^2^
Western	Pinar del Rio	500	0.42 (0.175–0.655)	24.7	0.78 (0.537–6.048)	19.5	4.83 (± 0.44)	22.45
	Matanzas	1000	0.2 (0.153–0.235)	11.76	0.9 (0.66–1.34)	22.5	1.9 (± 0.11)	13.63
	Santa Clara [Villa Clara]	700	0.152 (0.125–0.2)	8.94	0.4 (0.3–0.57)	10	3.12 (± 0.2)	9.22
Central	Cienfuegos	800	0.1 (0.077–0.125)	5.88	0.342 (0.251–0.54)	8.55	2.37 (± 0.14)	17.07
	Sancti Spíritus	700	0.114 (0.103–0.126)	6.7	0.307 (0.264–0.368)	7.67	2.97 (± 0.18)	2.64
	Camagüey	600	0.55 (0.263–1.189)	32.35	1.6 (0.866–52.612)	40	2.75 (± 0.24)	41.03
	Las Tunas	1000	0.016 (0.009–0.029)	0.94	0.067 (0.036–0.238)	1.67	2.08 (± 0.104)	107.8
Eastern	Holguín	600	0.175 (0.048–0.367)	10.3	0.53 (0.275–8.52)	13.25	2.67 (± 0.23)	38.19
	Santiago de Cuba	500	0.347 (0.14–0.6)	20.41	0.976 (0.573–8.33)	24.4	2.85 (± 0.3)	14.34
	Guantánamo	900	0.224 (0.163–0.313)	13.17	0.866 (0.566–1.707)	21.65	2.2 (± 0.13)	27.96
	New Orleans (susceptible strain)	720	0.017 (0.013–0.025)	–	0.04 (0.026–0.095)	-	3.37 (± 0.3)	10.12

^1^Number of larvae evaluated (*N*). ^2^ Lethal concentrations (LC_50_ and CL_90_) in mg/L. 95% confidence limits (CL) in parentheses. ^3^ Resistance ratio (RR_50_ and RR_90_): CL_50_ or CL_90_ *Ae. aegypti* colonies to be evaluated / CL_50_ or CL_90_ New Orleans susceptible strain. ^4^ Slope of the probit-log line: standard deviation (± SD) in parentheses. ^5^ Chi-square (*χ*^2^) of the probit test to determine goodness of fit

**Fig. 1:**
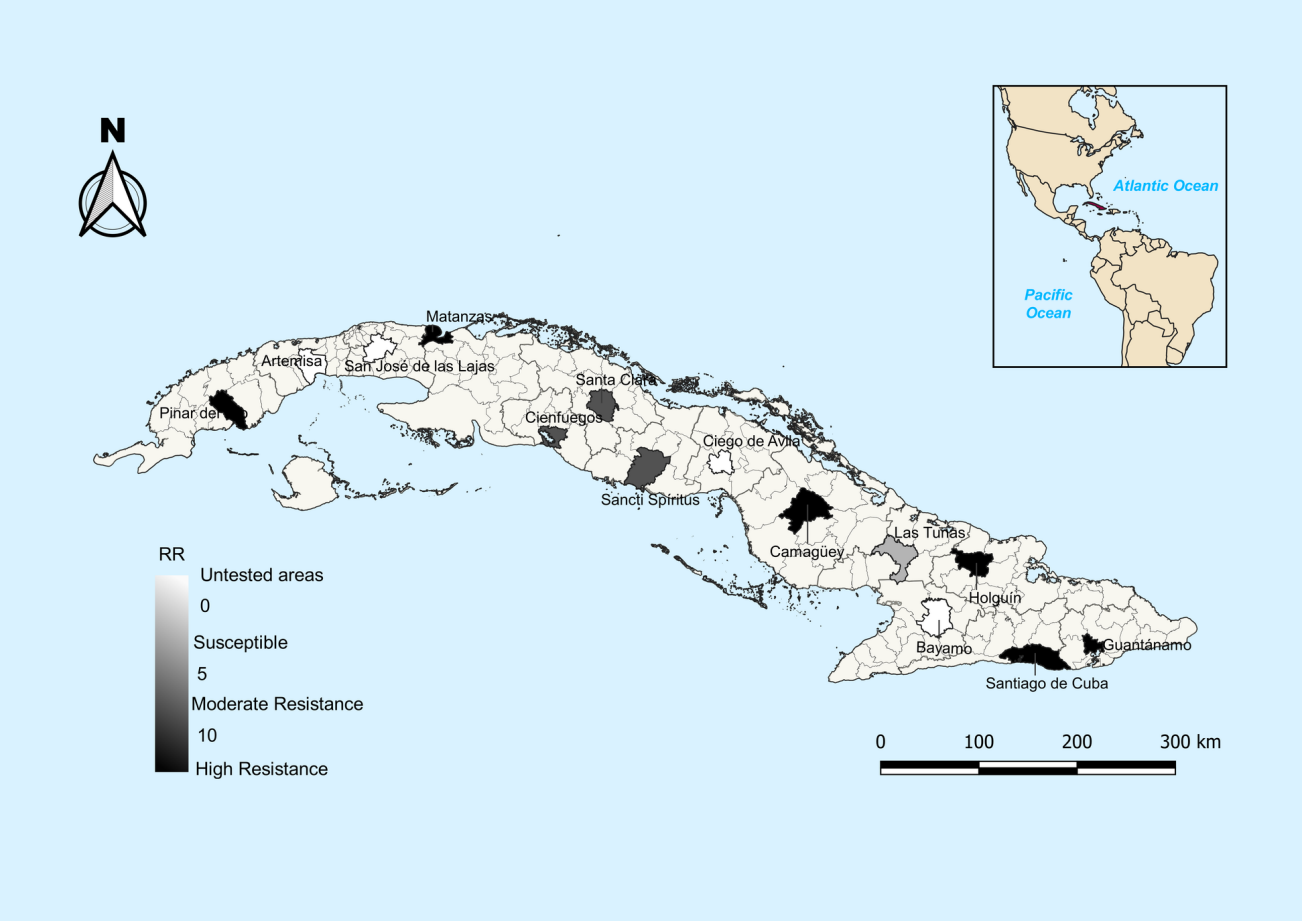
Geographical distribution of temephos susceptibility status in *Aedes aegypti* populations from western, central, and eastern regions of Cuba

Temephos susceptibility was presented by *Ae. aegypti* populations from Las Tunas, as reflected in light grey on the map (Fig. 1 and Table 2). Moderate resistance was presented by *Ae. aegypti* populations from Moderate resistance was presented by, Cienfuegos, and Sancti Spíritus, as reflected in dark grey on the map (Fig. 1 and Table 2). However, high temephos resistance was observed in the remaining mosquito populations tested, as reflected in black on the map (Fig. 1 and Table 2).

Pinar del Rio population showed the highest slope value (4.83) of the probit regression line (Table 1), which confirm its homogeneity to temephos resistance.

## Discussion

The intensive use of insecticides in Cuba, but also in other parts of the world, causes resistance in mosquito vectors, being one of the factors that reduce the success of vector control programs [30]. The emergence of insecticide resistance is a complex phenomenon that involves physiological, genetic, ecological, and behavioral factors combined with pesticide application [30].

As part of a strategic plan of the National Vector Control Program, this study was carried out by the Institute of Tropical Medicine “Pedro Kouri”-team to determine the geographical distribution of temephos resistance status in *Ae. aegypti* populations in Cuba.

In this study, most of Cuban *Ae. aegypti* populations tested from the western (Pinar del Rio and Matanzas), central (Camagüey), and eastern (Holguín, Santiago de Cuba, and Guantánamo) regions of Cuba showed high resistance to temephos (Fig. 1 and Table 2). These findings are consistent with the results of previous small-scale studies conducted between 1997–2014, where Pinar del Rio showed a resistance ratio (RR_50_ = 66.66) 2.7 times greater [19], while in Santiago de Cuba (RR_50_ = 26.94) it was slightly increased [18]. High resistance was also observed in other settings, outside Cuba, namely in Laos [31], Acre (Brazil) [32], Tamil Nadu (India) [33], Pernambuco (Brazil) [34], Martinique [35], and Bahia (Brazil) [36]. Moderate resistance was shown by *Ae. aegypti* populations from three provincial capitals of the central region (Fig. 1 and Table 2), such as Santa Clara [Villa Clara], Cienfuegos, and Sancti Spíritus. Only one *Ae. aegypti* population, namely from Las Tunas province, located in the eastern region of Cuba, showed temephos susceptibility. Several regions such as Tocantins (Brazil) [37], Paraná (Brazil) [38], Quindío (Colombia) [39], Delhi (India) [6], Sao Paulo, and northeast region of Brazil [40] have also reported moderate resistance. Temephos resistance reported in regions of Latin America such as Brazil [36] and Martinique [35], and Southeast Asia such as India [33] and Laos [31], is caused by the prolonged use of this larvicide by the National Programs for Dengue Vector Control. This has driven the selection of resistant *Ae. aegypti* populations in many parts of the world [4, 41]. This problem could affect the efficacy of mosquito larvae control.

Temephos susceptibility has been reported in La Dorada (Colombia), where a reduction in RR values was observed, which went from 13.27 in 2007 to 4.75 in 2011 [42]. This reversal of temephos resistance observed in *Ae. aegypti* populations from La Dorada could be owing to the fact that this larvicide was no longer used for 4 years. *Aedes aegypti* population from Las Tunas showed temephos susceptibility, which is reflected in the map (Fig. 1), where it can be observed how its resistance profile differed completely from the rest of the mosquito populations analyzed*.* It is curious that this result has been obtained in Las Tunas even though this larvicide has been used intensively since 1981 by the National Vector Control Program. It has been reported that different types of habitats, such as urban and rural areas, can provide insects with specific microclimates that influence their exposure and response to insecticides [43–47]. Several studies suggest that geographical barriers (mountainous areas or water bodies) can restrict genetic flow between insect populations, leading to different resistance profiles among populations as well [43–47]. It is also suggested that mosquito populations in different geographical regions may present heterogeneity in resistance profiles owing to climatic conditions such as temperature changes [43–47]. It would be appropriate to carry out a population genetics study to determine if there is genetic variability in the *Ae. aegypti* populations from Cuba.

It has been reported that there are mosquito populations that evolve faster than other populations in terms of insecticide resistance development [48]. Insecticide resistance evolves relatively rapidly and is primarily a biochemical phenomenon [49]. It is based on the selection of genes that code for enzymes involved in insecticide detoxification, or that confer insensitivity at their target sites [50]. Individuals carrying resistant genes are initially rare in natural populations. However, as selective pressure increases, these individuals constitute an increasing proportion of insect populations, while their susceptible counterparts are progressively eliminated. This accelerated evolution of resistance implies additional costs that are more marked in some insect species, which have a slower evolution than others, where the costs are not as pronounced [51]. The overexpression of detoxifying enzymes occurs at the cost of the decrease of other important traits that are associated with insect growth, maturation, longevity, and reproduction rates [50].

The results of this study were similar to those obtained in Peru [52], where *Ae. aegypti* populations from three regions had different susceptibility levels to temephos.

The findings warrant further analyses in resistant *Ae. aegypti* populations from western, central, and eastern regions of Cuba to determine the mechanisms associated with temephos resistance. The limitation of this study was the lack of equipment and reagents (synergists) necessary to identify the metabolic enzymes involved in resistance to this larvicide.

The increase in the slope value (Table 2) of the probit regression line confirms homogeneity to temephos resistance in Pinar del Rio populations. It would be appropriate to carry out a study about population genetics to elucidate if there are genetic differences among *Ae. aegypti* populations. In the probit test, the slope represents the relationship between insecticide dose and mosquito mortality. A low slope value (≈0) implies greater uncertainty in the LC_50_ estimate, which may indicate heterogeneity of resistance or susceptibility in mosquito populations. However, a high slope value implies greater precision in the LC_50_ estimate since small variations in dose cause large changes in mortality. This may indicate homogeneity of resistance or susceptibility in populations.

Temephos resistance was shown in 9 out of 10 provinces tested, representing 56.25% of all provinces in Cuba, which is a major concern for the National *Aedes aegypti* Control Program. It would be appropriate to evaluate temephos susceptibility status in mosquito populations in the rest of the country’s provinces to assess the impact of national control actions. Some authors have reported a significant decrease in the residual effect of temephos in highly resistant mosquito populations being effective for a period of 13 days and susceptible populations for 18 days [22].

These results showed an imperative need to implement new integrated vector control strategies at a national scale, such as those using alternative insecticides on the basis of *Bacillus thuringiensis var israelensis* (*Bti*) or pyriproxyfen (insect growth inhibitor) to avoid temephos resistance development in the *Ae. aegypti* populations of Cuba. A successful strategy carried out in Boyeros municipality (Havana city) showed how resistance levels decreased in mosquito populations when temephos application was suspended and replaced by *Bti* [53]. Similar results were obtained in Brazil using this microbial control agent and using growth inhibitors [36, 54, 55]. It has also been shown that temephos susceptibility can be recovered in *Ae. aegypti* lab strains because its metabolic resistance mechanisms are reversed when its use is discontinued [56, 57]. Preferably, these strategies could be carried out by promoting insecticide rotation policies to preserve temephos effectiveness.

## Conclusions

Most *Ae. aegypti* populations tested from the western, central, and eastern regions of Cuba showed spatial homogeneity of temephos resistance, which could be owing to the intensive and prolonged use of this larvicide since 1981. Temephos resistance detected in most of mosquito populations tested warrants further analyses to determine their resistance mechanisms. These findings show the urgent need to implement insecticide rotation policies, such as the use of alternative products based on *Bti* or insect growth inhibitors to avoid or reverse an increase in resistance to temephos in mosquito populations.

## Data Availability

Data supporting the main conclusions of this study are included in the manuscript.
